# 

**Published:** 1899-04-01

**Authors:** 


### The Hospital

A JOURNAL OF

THE MEDICAL SCIENCES AND HOSPITAL ADMINISTRATION,

INCLUDING

A SPECIAL SECTION FOR NURSES.

EDITED BY

SIR HENRY BURDETT, K.C.B.,

AND

SOLOMON C. SMITH, M.D.

IN TWO VOLUMES ANNUALLY.

VOLUME XXVI.

April to September, 1899

LIBRARY

SURGEON GENERAL'S OFFICE

AUG -8 1900

165962

London:
PUBLISHED BY THE SCIENTIFIC PRESS (LIMITED), AT THE HOSPITAL BUILDING,
28 & 29, SOUTHAMPTON STREET, STRAND, W.C.

MDCCCXCIX.

WI
NO69X

### INDEX TO VOL. XXVI., 1899.

*⁎* Articles under the general heading *Hospital Clinics and Medical Progress* are distinguished by the initial (C.) for *Hospital Clinics*, and by (P.) for the rest of the series. (W.) distinguishes the *Institutional Workshop* articles, and (A.) the *Annotations*.

Abdomen, General Surgery of the (P.), 214
Abdominal Wounds (P.), 214
Aberdeen Asylum New Hospital (W.), 336
Abscess of the Liver (P.), 229
Acetanilid (C.), 228
Acre of Land as an Old Age Pension, An (A.), 3
Acromegaly (P.), 349
Actinomycosis, Pulmonary (P.), 212
Addison's Disease (P.), 348
Adenitis, Cervical, Treatment of (C.), 364
Adenoids and Enuresis (C.), 61
After-Care Association for the Insane (W.), 152
Agoraphobia (P.), 64
Air Space Question, The (A.), 257
Airol (P.), 383
Alcoholism, Acute (P.), 297
Alcoholism, and General Paralysis (W.), 152
Alkaptonuria (C.), 128
Alps, Holidays in the (A.), 223
Anæmia, Pernicious (P.), 330
Anæsthetics, Progress in (P.), 279, 298
Aneurism, Aortic (P.), 27
Ankylosis and its Treatment (C.), 209, 224
Antero-lateral Ascending Tract (P.), 63
Antipyrin (P.), 386
Antitoxin and Diphtheritic Paralysis (C.), 381
Antitoxins for Tuberculosis (P.), 81
Antituberculous Serum (C.), 61
Aphasia (C.), 95 (P.), 262
Apoplexy and First Aid (A.), 175
Appendages, Result of Removal of the (C.), 77
Appendicitis (P.), 247, 382
Arthrotomy for the Relief of Pain (C.), 145
Artificial Respiration, Schultze's Method, 277
Ascites of Cirrhosis, Operation for (C.), 180
Ascites, Hæmorrhagic (P.), 247
Asepsis in Surgery (P.), 425
Asthma (P.), 182, 196
Astragalus, Excision of (P.), 406
Ataxy, The Clinical Relations of (C.), 110
Atrophy, Acute Yellow (P.), 230
Attractive Mortuaries (A.), 190
Australian Inebriates' Bill (W.), 411
Auto-Intoxications (P.), 246
Bacillus ærogenes capsulatus (P.), 231
Bacteria of Pregnant and Puerperal Vagina (P.), 166
Bacteria, The Resistance of, to Cold (A.), 223
Bacteriology, Progress in (P.), 80, 230
Bedford County Hospital, Opening of the (W.), 217
Blood, Diseases of the (P.), 330, 348
Blood, Examination of the (P.), 348
Boarding Ships as Board Schools, 282
Book World of Medicine, The (*Books, Magazines, and Pamphlets Reviewed*)
Aikman. Milk: its Nature and Composition, 216
Allbutt. A System of Medicine, 100, 150, 408
Baker. Parish Problems, 66
Bishop. Diseases of the Ear, Nose, and Throat, 426
Bray. The Cry of the Children, 66
Bruce. Materia Medica and Therapeutics, 216
Burdett's Hospitals and Charities, 462
Church Lads' Brigades Medical Corps, Manual for the, 462
City of London Directory for 1899, 82
Code of Rules for the Prevention of Infectious Diseases, 50
Cox and Stokes. The Pocket Pharmacopœia, 266
Crile. An Experimental Research into Surgical Shock, 82
Dalton. Gleet and Chronic Diseases of the Urethra, 462
Diseases of Animals Act: Report, 387
Dukes. Remedies for Needless Injuries to Children, 232
Edinger. (Trans. by Hall.) Anatomy of the Central Nervous System, 300
Elder and Fowler. Diseases of Children, 250
Evans. Golden Rules of Medical Practice, 334
Fitzgerald. Pyorrhœa Alveolaris, 319
Foster. Claude Bernard, 300
Frazer. Selby: A Pathological Morality, 387
Goodhart and Still. Diseases of Children, 28
Grandin and Jarman. Text-book of Practical Obstetrics, 426
Hawkins-Ambler. Saline Injections in Abdominal Operations, 82
Hawkins-Ambler. The Commonwealth of the Body, 352
Hayward. The Construction of Hospitals for Consumption, 200
Herschell. Constipation and its Modern Treatment, 216, 462
Book World of Medicine (*continued*).
Hill. Manual of Physiology, 387
Hovis Cycle Road Map, 66
Hueppe. (Trans. by Jordan.) Principles of Bacteriology, 50
Jellett. Short Practice of Midwifery, 334
Kingscote. Asthma, 462
McCaw. Aids to the Treatment of the Diseases of Children, 319
Medical Annual and Practitioners' Index, 66
Nadkari. Essentials of Modern Treatment of Disease, 387
Newsholme. Elements of Vital Statistics, 282
Ostrom. Massage and the Original Swedish Movements, 319
Park. An Epitome of the History of Medicine, 352
Pearmain and Moor. Chemical and Biological Analysis of Water, 82
Pharmacopœia of Guy's Hospital, 352
Pritchard. London and Londoners, 1899, 266
Reeves. Intestinal Obstruction, 370
Reynolds-Ball. Mediterranean Health Resorts, 28
Savill. Clinical Lectures on Neurasthenia, 232
Scarfield. The Use of Tuberculin, 200
Shaw. Golden Rules of Psychiatry, 352
Shelley. Chats About the Microscope, 334
Smith. Natural Mineral Waters of Harrogate, 250
Tebb. A Century of Vaccination, 250
Walker. Introduction to Dermatology, 319
Webb. Harry Ingleby, Surgeon, 66
Whitelegge. Hygiene and Public Health, 200
Wide. Medical Gymnastics, 266
British Association, The (A.), 434
British Medical Association Meeting (C.), 294, 312
Bromide of Potassium (P.), 296
Bronchi, Foreign Bodies in the (P.), 196
Bronchitis (P.), 181
Burial Laws, Our (A.), 361
Buxton (W.), 201
Cæsarian Section (P.), 183
Cancer (P.), 230
Cancer, What can Surgery do for (P.), 330
Cancer, Ætiology of (C.), 60, (P.), 298
Cancer of the Breast, Dissemination of (P.), 316
Cancer of the Breast, Oophorectomy for (P.), 331
Cancer, The Cause of (C.), 277
Cancer Fields (C.), 59, Houses (C.), 60
Cancer, Inoperable (P.), 331
Cancer, Mammary (P.), 316
Cancer, The Origin of (C.), 7
Cancer of the Stomach (P.), 146
Carcinoma, Pancreatic (P.), 230
Case-Books, The Ownership of Hospital (A.), 36
Catarrh, Chronic (P.), 146
Cerebral Activity and Sleep (P.), 63
Cerebral and Cerebellar Abscesses (P.), 459
Cerebro-Spinal Fever (C.), 228
Cerebro-Spinal Fluid (P.), 63
Cervix Uteri, Malignant Disease of the (P.), 165
Charitable Dispensaries of Bengal, The (W.), 52
Chelsea Workhouse Infirmary (W.), 338
Cheyne-Stokes Respiration (P.), 212
Chigoe, The (A.), 175
Child-bearing, Diseases Resulting from (C.), 177
Children, Diseases of (P.), 213
Chorea (P.), 296, 439
Chorea, Chloral Sleep in the Treatment of (C.), 95
Cirrhosis of the Liver (P.), 214, 229, 247
Civilisation and Typhoid Fever (A.), 18
Climate, Sources of Fallacy in Regard to Treatment by (C.), 179
Clinical Society (C.), 179
Club Practice and Expensive Drugs (A), 241
Cocainising the Spinal Cord (C.), 261
Cœliotomy, Vaginal (P.), 147
Coleman v. Caldwell, 55
Collecting Books (A.), 399
Collective Research (A.), 241
Colon, Idiopathic Dilatation of the (P.), 246
Colonial Nursing Association (W.), 303
Colotomy, Right, for the Relief of Pain (C.), 145
Colour-blind Sailors (A.), 453
Common Lodging House, What is a? (A.), 343
Companies, Medical Practice by (C.), 23
Compulsory Removal and Detention of Fever Patients (W.), 356
Concentrated Foods (C.), 210
Consciousness and Anæsthetics (A.), 207
Consulting Institute, A (C.), 127
Consumption, The Cure of, 140
Consumption, The Open-air Treatment of (C.), 276
Consumption, Rational Treatment of (C.), 178
Consumption, The Voluntary Notification of, 222
Consumptive Patients at Natal (A.), 91
"Contagion" or "Infection," 221
Coroner's Inquest without a Jury (A.), 241
Corporal Punishment for Criminals, 67
Cottage Hospitals. *See p.* 267
Cottage Hospitals and Sick Pay (W.), 254
Cough, Causes of, in Children (C.), 365
Coughs, Treatment of with Heroin (P.), 181
Cousins (Dr.) on the Century's Progress, 309
Coxa Vara (P.), 97
Creasote Inunction in Malarial Fever (C.), 278
Cremation, 35
Cretinism, Sporadic, Thyroid Treatment (P.), 62
Cysts, Hydatid (P.) 248
Dangerous Industries, 256
Death Certificates in Coroners' Cases (A.), 378
Death from Drowning (A.), 19
Dermatitis, Desquamative (P.), 407
Diarrhœa, Autumnal (C.), 229
Diet in the Typhoid State (C.), 210
Digestive Organs, Diseases of the (P.), 245
Diphtheria (C.), 455
Diphtheria, Bacteriological Diagnosis of (C.), 211
Diphtheria, Results of Antitoxin Treatment in Germany (C.), 227
Disease and Insanity, 323
Disinfection by Steam (A.), 3
Doctor as a Carrier of Disease, The (A.), 309
Drink and Heredity, 290
Drop-foot, Tendon Grafting for (P.), 406
Dundee, Victoria Hospital (W.), 428
Duodenal Ulceration (P.), 245
Dysentery (P.), 246
Dyspepsia, Nervous (P.), 146
Eastbourne, The Tragedy at (A.), 453
Eclampsia, Puerperal, Saline Injections in (C.), 245
Eczema (P.), 79, 385, in Infants (P.), 422
Emigration of Pauper Children, The (A.), 343
Emphysema (P.), 196
Emphysema, Pulmonary, The Nature of (C.), 6
Empyema of the Thorax (P.), 439
Endocarditis, Malignant (P.), 26
Enteritis, Membranous (P.), 246
Enuresis, Adenoids and (C.), 61
Enuresis, Treatment of, by Distension (P.), 440
Epilepsy (P.), 96, 97, 295
Epilepsy, Auto-intoxication in (P.), 96
Epilepsy, Bromides in the Treatment of (C.), 347
Epileptic Lunatics (C.), 228
Erysipelas (P.), 98
Erythromelalgia (P.), 130
Essex Water Supply, Dr. Thresh on (A.), 325
Extra-Dural Abscess (P.), 423
Extra-Uterine Pregnancy (P.), 147
Eye Hospital, The Oldest in England (W.), 302
Eyesight, Trade Risks to (A.), 324
Eye-Strain and the Rest Cure (C.), 245
Facial Paralysis of Aural Origin (P.), 297
Famine and Infant Mortality, 141
Fat, Diet for the (C.), 45
Feeding Bottles, Long Tube (C.), 228
Femur, Traumatic Epiphysial Separation of Head of the (P.), 385
Fevers (P.), 8, 24, 113, 366, 404
Field Surgery in South Africa, 451
Filarial Periodicity (C.), 421
Finger in Every Pie, A (A.), 159
Fleeing from Disease (A.), 91
Fly-proof House, A (A.), 91
Food Poisoning (P.), 148
Food Preservatives and the Food and Drugs Amendment Act (A.), 124
Fractures and Dislocations (P.), 9, 27, 48, 440
Fractures, Simple (C.), 193
Frankland, the late Sir Edward (A.), 343
Funeral Scarves and Secret Commissions (A.), 435
Gastric Arteries, Minute Erosion of (P.), 131
Gall Tracts, Operation on the (P.), 248
General Surgery (P.), 9, 27, 48, 425, 440
Genito-Urinary Surgery (P), 64
Girl from the Union, The, 12
Goitre, Exophthalmic, Surgical Treatment (P.), 63
Gonococcus Infection, Diagnosis of (C.), 401
Gonococcus Joint Disease (P.), 213
Gonorrhœa (P.), 65, 350
Gross Fraud upon the Poor, A (A.), 37
Gruel as an Infants' Food (C.), 347
Guiacol Vapour Baths in Bronchiectasis (C.), 295
Gunshot Wounds of the Abdomen (C.), 328
Gynæcology (P.), 132, 147, 165, 332, 350, 368
Hæmatomyelia, Primary Focal (P.), 112

Hæmatura, Hæmorrhage in (C.), 75
Hanwell Ophthalmic School, The (A.), 57
Head-nodding in Infants (P.), 96
Health Resort, Choice of a (A.), 143
Health Resorts, Notes on (W.), 335
Heart and Circulation, Diseases of the (P.), 25
Hernia (P.), 164, 263, 279
Hernia, Femoral, Radical Cure of (P.), 263
Hernia, Inguinal, in Childhood (P.), 214
Hernia, Inguinal, Radical Cure of (P.), 164
Hernia, Interstitial (P.), 264
Heroin. *See p.* 181
Herpes (P.), 98
Hip, Congenital Dislocation of the (P.), 422
Hip, Modern Treatment of Congenital Displacement of the (P.), 384
Hodgkins' Disease (P.), 330
"Hooligan" Girl, The (A.), 379
Hospital Administration (W.), 169, 410
Hospital Construction (W.), 68, 102, 117, 336, 355, 410, 428, 445, 465
Hospital Construction, The Ever-growing Cost of (W.), 252
Hospital Festivals, Meetings, &c. (W.), 14, 30, 53, 69, 85, 103, 120, 134, 154, 171, 185, 202, 219, 234, 269, 2[illegible]4, 321
Hospital Laundries (W.), 354
Hospital Milk (A.), 175
Hospital Sewage, The Treatment of (W.), 68
Hospitals, Temporary (W.), 411
House of Commons, The, and the Army Medical Officers (A.), 191
Housing of the Working Classes (P.), 460
Hyperchlorydia (P.), 146
Hypertrophy, Prostatic (P.), 64
Hysterectomy as a Conservative Operation (P.), 368
"Immediate Personal Supervision," 239
Incontinence of Fæces, Nervous (P.), 422
Inebriates Act, The (C.), 244
Ir[illegible]ts, Rapid or Sudden Death in, from Unsuspected Causes (C.), 275
Infectious Cases in General Hospitals (W.), 373
Infectious Diseases, Methods of Isolation (C.), 39
Infectious Diseases, The Notification of (W.), 430
[illegible]finitely Little, The (A.), 73
[illegible]luence of One Disease upon Another (C.), 260
[illegible]fluenza, (P.), 8
[illegible]nsane, The Punishment of the, 416
[illegible]nsane, The Story of the Insane from Year to Year, 84, 102, 170, 286, 338, 355, 375, 447
Insanity, The Borderland of, 450
Insanity, A Study in, 449
Insects and the Dissemination of Diseases (C.), 420
Insomnia, 271
Inspection of Food a Remunerative Undertaking (A.), 107
Institutional Questions (W.), 356, 375, 413, 431, 447, 465
International Council of Women (A.), 107
Intestinal Bacteria, Importance of, in Digestion (C.), 438
Intestines, Surgery of the (P.), 367
Iodide of Potassium in Cases with Obstructed Bronchi (C.), 96
Iritis, Treatment of (C.), 260
Juvenile Delinquents, 151
Knee Joint, Exploration of the (C.), 314
Lamont (Dr.), The Case of, 272
Lamps, The Right Kind of (A.), 207
Law's Delays, The, 301
Leek Cottage Hospital (W.), 410
Leprosy (P.), 99
Leprosy in the Baltic Provinces (W.), 446
Leucæmia, Acute Splenic (P.), 315
Leucocytosis and Eosinophilia (C.), 22
Liabilities for the Treatment of Pay Patients in Hospitals (W.), 374
Lichen Annularis (P.), 407
Licking the Fingers (A.), 257
Limitation of Families in County Council Dwellings (A.), 56
Lipomata (P.), 297
Little White Slaves (A.), 125
Liver and Gall Bladder, Surgery of the (P.), 247
Liver and Pancreas, Diseases of the (P.), 228
Liverpool Hospital for Cancer (W.), 169
Liverpool Fever Hospital (A.), 435
Lobar-Pneumonia in Acute Specific Fever (C.), 59
Lobar-Pneumonia, A Case of (C.), 109, 127
London County Council, The, 291
London Fever Hospital Nurses' Home (W.), 102
London Polyclinic, The (A.), 207
London School of Tropical Medicine, 89
London, University of (C.), 261
Lower Extremity, Fractures, &c. (P.), 48
Lumbar Puncture (C.), 327
Lunacy Bill, The, 90
Lunacy in the Central Provinces of India (W.), 85
Lunatic at Large, The, 452
Lupus (P.), 79, 98, 385
Madras, Hospitals and Dispensaries in (W.), 287
Malaria (P.), 25, 113, 404
Malaria Problem, The (C.), 328
Marriage in the Case of Woman (C.), 143
Marriage of the Unfit, 51
Masseuses, The Troubles of the, 139
Mastoid Disease (P.), 423
Measles, Koplik's Sign in (C.), 211
"Medical Aid" and "Friendly" Societies, 189
Medical Advertising on the Continent, 73
Medical Council, The General (C.), 180, 195
Medical Council and "Pious Opinions" (A.), 190
Medical Curriculum, The, 389
Medical Defence and Medical Aid (A.), 309
Medical Degrees and other Qualifications, 391
Medical Fees in Cottage Hospitals, 69
Medical Graduates' College and Polyclinic (A.), 36
Medical Institutions of Calcutta, The (W.), 52
Medical Officers and Workhouse Nursing (C.), 245
Medical Profession, The, 377
Medical Schools, The, 393
Medical Strike, A Real, 397
Medical, Surgical, and Hygienic Exhibition (W.), 152
Men and Machinery, 29
Meningitis (P.), 459
Meningitis, Cerebro-Spinal (C.), 404
Meningitis, Kernig's Sign in (P.), 422
Meningitis, Simple Posterior Basic, of Infants (P.), 231
Metropolitan Asylums Board (A.), 37, 291
Metropolitan Hospital Sunday Fund (W.), 305
Metropolitan Hospital Sunday Fund, A Brief History of the (W.), 429
Metropolitan Provident Medical Association, The (A.), 191
Micawberism (C.), 110
Microbes in the Air of Schools (A.), 417
Middle Ear, Suppuration of the (P.), 423
Midwifery, Aseptic, An Address on (C.), 419, 437
Milk from the Aldershot Sewage Farm (A.), 223
Mistress, The Education of the, 463
Modern Sociology, 12, 67, 83, 101, 116, 151, 168, 233, 251, 267, 283, 301, 353, 371, 388, 409, 427, 444, 463
Morris (Mr. Malcolm), Tuberculosis and the College of Physicians (A.), 379
Mosquito, The "Domestic," 315
Mosquito Hunt at Sierra Leone, The (A.), 434
Municipal Milk (A.), 361
Musculo-Spinal Nerve, The, in Fracture of the Humerus (C.), 77
Muzzling and Rabies, 205
Mycosis Fungoides (P.), 406
Myopathy (C.), 195
Myositis, Progressive Ossifying (P.), 212
Myxœdema (P.), 47
Naked Lights (A.), 57
Naval Medical Service, The, 415
Needle, How to Thread a (C.), 439
Nephritis Dependent on Gastro-Enteritis (P.), 214
Nerve Training, 388
Nervous Disease, Death from Functional (C.), 46, 63
Nervous System, Diseases of the (P.), 93, 111, 129
Neuritis, Peripheral, Retrobulbar (P.), 296
Neurology (P.), 262, 278, 295
Neurotic Ulcers of the Mouth (P.), 297
New Appliances and Things Medical (W.), 16, 49, 65, 115, 133, 149, 167, 184, 199, 249, 265, 281, 299, 318, 333, 351, 369, 386, 407, 443, 461
Night Terrors and Nightmare (C.), 364, 440
Non-Nitrogenous Diet in Epilepsy (A.), 343
North-Western Fever Hospital, The, 324, 357
Notes (W.), 268, 284, 320, 338, 355, 372, 412, 446
Notes and News, 4, 20, 38, 58, 74, 92, 110, 126, 142, 160, 176, 192, 208, 224, 242, 258, 274, 292, 310, 326, 344, 362, 380, 400, 418, 436, 454
Notes on Health Resorts (W.), 201
Obstetrics, Progress in (P.), 166, 182
Obstructing Removal of Patients to Fever Hospitals (W.), 153
Œdema (C.), 22
Operation, A Single-handed (W.), 375
Ophthalmia neonatorum (P.), 248
Ophthalmia, Sympathetic (C.), 458
Ophthalmology, Progress in (P.), 248
Ophthalmoscopy in Diagnosis (P.), 460
Organisms of Disease (A.), 125
Orthopædic Surgery (P.), 97, 383, 405, 424
Otitis Media, Treatment (P.), 460
Otology (P.), 423, 442, 459
Ovarian Tumours, Torsion of Pedicle of (P.), 133
Overcrowding in St. Pancras, 173
Overcrowding of the Underclad, The (A.), 378
Paddington Green Children's Hospital (W.), 117
Pædiatrics (P.), 422, 439
Painful Experiments on School Children (A.), 257
Pancreas, Calculi of the (P.), 230
Pancreatic Carcinoma (P.), 230
Paralysis Agitans (P.), 296
Patented Germs (A.), 417
Payments by Patients (W.), 268
Peace Congress, The (A.), 191
Peculiar People, The (A.), 117
Pediculosis (P.), 407
Pemphigus of Conjunctiva (P.), 248
Pericarditis (P.), 25, 213
Periscope of Dermatology (P.), 79, 98, 385, 406
Pessaries, Use and Abuse of (P.), 333
Pharmacology, The Teaching of (A.), 343
Phosgene Gas (C.), 227
Phthisis (P.), 78
Phthisis, Fibroid, in the Gold Mines (C.), 439
Phthisis with Peculiar Physical Signs (P.), 198
Phthisis, Pretuberculous Stage of (P.), 198
Physiology in Education, 71
Picture in Bumbledom (A.), 57
Pity the Poor Landlord, 101
Placenta Prævia, A New and Safe Method of Delivery in (C.), 363
Plague, The (A.), 342
Plague, Precautions against (C.), 402
Plague, Progress of the (A.), 159
Plantar Reflex in Infants, The (P.), 440
Play Acting as a Substitute for Sermons, 267
Pneumonia (P.), 196
Pneumonia in Infants (P.), 126
Politics and Profession, 291
Polyclinic, The (A.), 435
Polymyositis (P.), 129
Poor of Denmark, The, 116
Poor Laws, The, 359
Porro's Operation, A (C.), 46
Portsmouth, New Royal, Hospital (W.), 234
Pott's Disease (P.), 97
Powell (Sir R. Douglas) on Recent Advances in Practical Medicine (C.), 313
Practical Departments (W.), 16
Practical Points (W.), 254, 287
Pregnancy and Factory Work, 168
Preventive Inoculation, 253
Preventive Treatment in the Diseases of Women (C.), 161, 177, 193
Prince of Wales's Hospital Fund (W.), 119
Princess Mary Convalescent Home (W.), 465
Prickly Heat (C.), 314
Private Hospitals and Nursing Homes, 206
Prognosis in Operations for Appendicitis (C.), 162
Protargol (P.), 248
Pseudo-Tuberculosis (P.), 81; (C.), 146
Psilosis or Sprue (C.), 420
Puerperal Eclampsia (P.), 184, 245
Puerperal Fever, The Treatment of (P.), 182
Pulmonary Abscess (P.), 197
Pulmonary Regurgitation (P.), 26
Pyo-pneumothorax (P.), 197
Queen Charlotte's Nurses' Home (W.), 284
Queue System at Hospitals, The (W.), 52
Rabies (P.), 115
Rabies, Prophylactic Cauterisation in (C.), 23
Rating of Charitable Institutions (A.), 273
Real Physicians (A.), 309
Reciprocity, 341
Rectum and Anus, Surgery of the (P.), 198
Red Cross Societies in War, 105
Renal Calculi, Detection of, by Radiography (C.), 162
Respiratory System, Diseases of the (P.), 78, 181, 196, 212
Retinal Changes (P.), 112
Rio Tinto Hospital (W.), 410
Rodent Ulcer, The Superficial Form of (C.), 365
Roentgen Rays (C.), 381, (P.), 406
Roentgen Rays and Colour Blindness (C.), 128
Royal College of Surgeons, The (C.), 261
Royal Colleges, Privileges of the (C.), 194
St. Mary, Islington, New Infirmary (W.), 68
St. Thomas's Hospital, Opening of the New Accident Ward (W.), 119
Sale of Food and Drugs Bill, The, 289
Sale of Sanitary Indulgences (A.), 159
Salvation Army, The (A.), 73
Sanitary Record of House Conditions, A (C.), 403
Scarlet Fever, The Microbe of (A.), 3
Scarlet Fever, Spread by Milk (A.), 141
Scarlet Fever in the Tropics (C.), 421
School Children and Their Industries, 106
School Space (A.), 325
Secret Commissions, 398
Self-conceit (A.), 452
Sensation, Disturbances of (P.), 111
Septic Infection of Ovarian Cysts (P.), 165
Serum Treatment of Fever after Delivery (C.), 328
Seven-day Newspapers, 123
Sinus Thrombosis (P.), 442
Skin Grafting (C.), 458
Small-pox (P.), 8
Small-pox, The Aerial Convection of (A.), 273
Snake Bite (P.), 114
Soil and Disease (P.), 317
Song Birds as a Source of Tuberculosis (C.), 144
Speaking Tubes (A.), 19
Specialism (C.), 227
Specialism, The Nemesis of, 2
Speed in Operating, A Plea for (C.), 363
Spina Bifida (P.), 131
Spine, Deformities of the (C.), 243, 259
Spine, Forcible Rectification of the (P.), 383
Spine, Injuries of the (P.), 132
Spine, Lateral Curvature of the (P.), 98
Spine, Lateral Deviation of the, and Pes Canis in Friedreich's Ataxia (P.), 384
Spine, Surgery of the (P.), 131
Spirit of Soap as an Antiseptic (C.), 295
Spontaneous Fracture in Factory Workers (C.), 348
Squint, At What Age to Operate for (C.), 457
Standardisation, Physiological, of Drugs (C.), 438
State Medicine, Progress in (P.), 317, 460
Steel, Removal of, by Electro-Magnet (P.), 249
Stomach, Diseases of the (P.), 130, 146
Stomach, Methods of Examining the (C.), 5
Stomach, On Removal of the (C.), 23
Stomach, Surgery of the (P.), 215
Student of Medicine, The, 54, 88, 254, 288, 322, 340, 358, 376, 414, 432, 466

Suicide, The Ethics of, 371, 409, 427
Sulphocarbolate of Soda in Chorea (P.), 439
Summer Diarrhœa (C.), 244
Superannuation Under the Poor Law, 70
Superfluous Hairs, The Treatment of (C.), 163
Suppuration, Pathology of (C.), 293, 311
Surgery. What Can Surgery Do? 60
Surgical Cleanliness, 56 *and see p.* 156
Syphilis (P.), 65
Syphilis and Hydrocephalus (P.), 422
Syphilis, Prevention and Treatment of (C.), 347
Syphilitic Deafness (P.), 459
Syphilitic Ulceration of the Stomach (P), 131
Tabes, Experimental (P.), 129
Tabes and Syphilis (P.), 129
Tachycardia, Essential Paroxysmal (P.), 26
Talipes Equino-Varus, Congenital (P.), 406, 425
Taxis, The Use of, in Reducing Herniæ (C.), 93
Temperance in the Army, 174
Temperature and Health, 307
Tetanus (P.), 114
Tetanus and Tetanus Antitoxin (P.), 366
Tetranitrate of Erythrol, 54
Therapeutic Notes, 407, 439
Thiocol in Phthisis (P.), 212
Thread Worms (P.), 213
Thyroid Gland, Diseases of the (P.), 47, 62
Thyroid Treatment for Children (P.), 440
Tibia, Hypertrophy of the (P.), 405
Tinned Foods (P.), 149
Tobacco Bill, The National, 17
Toluidin Blue (P.), 249
Toxæmic Delirium in Heart Disease (P.), 26
Toxicology, Progress in (P.), 148
Tracheotomy in Scarlet Fever (C.), 294
Training for Life Insurance (A.), 435
Transfusion in Diabetic Coma (C.), 381
Treatment of Symptoms, The, 158
Trichinosis Sporadic (C.), 22
Tropical Fevers (P.), 24
Tubercular Ear Disease (P.), 460
Tuberculin (C.), 163, (P.), 212
Tuberculosis (P.), 80
Tuberculosis, Climate in Treatment of (C.), 178
Tuberculosis, The Congress on, 157
Tuberculosis, as Discussed at Portsmouth, 360
Tuberculosis and Milk (P.), 212
Tuberculosis among Sailors (A.), 19
Tuberculosis and Strains (A.), 107
Tumours (P.), 262, 297, 330, 349
Tumours, The Diagnosis of (C.), 21
Twang of the Yankee, The (C.), 261
Typhoid (P.), 8, 24
Typhoid Fever (P.), 366
Typhoid Fever in American Cities (A.), 361
Typhoid Fever, Sick Room Infection in (A.), 399
Typhoid Fever, Vagaries of (C.), 22
Typhus Fever (C.), 76
Ulcers of the Tongue, Some (C.), 345
University Extension Lectures for Medical Men (A.), 273
Unqualified Dispensers (A.), 124
Upper Extremity Fractures, &c. ,(P.), 10, 27, 440
Urine and Fæces as Vehicles of Typhoid Infection (C.), 144
Uterine Fibroids, Operative Treatment (P.), 123
Uterine Hæmorrhage as affected by Altitude (P.), 166
Uterine Hæmorrhage, The Treatment of (P.), 369
Uterus, Inversion of the (C.), 277
Uterus, Malignant Disease of the (P.), 369
Uterus, Prolapse of, New Treatment (P.), 166
Uterus, Rupture of the (C.), 95
Vaccination, Aseptic, in New York (C.), 458
Vaccination in Germany (W.), 464
Vaccination and Insurance (A.), 37
Vaccination Officer, Duties of the (A.), 379
Vaccination and Small-pox in the U.S. Army, 329
Vaccination, "Successful" or "Efficient," 73
Varicose Veins in the Stomach (P.), 131
Ventilation (W.), 13
Veil, Use of the, by Operating Surgeons (C.), 128
Vesical calculi, Encysted (C.), 128
Vestry's Perversity, A (A.), 141
Visitation of Anglican Convents, The, 83
War in Cuba, Sanitary Lessons of the (C.), 226
War, Pestilence, and Famine, 434
Waste of Town Water (A.), 417
Water as a Medicine (C.), 210
Water for Sanitary Purposes (A.), 399
Who is to Certify (A.), 18
Williamsport City Hospital (W.), 445
Women can do, What, 353
Women, Diseases of, Preventive Treatment in (C.), 161, 177, 193
Women's Congress, The, 240
Workhouse Infirmaries and Consultants (W.), 169

### THE SPECIAL SECTION FOR NURSES.

Across the Seas, 23, 35, 66, 89, 116, 271, 284, 296, 310, 325
American Letter, Our, 67
Antiseptics and Operations, 48, 61, 73
Army Nursing Service Reserve, The, 87
Association of Asylum Workers, The, 8
Association for Promoting the Compulsory Registration of Midwives, Annual Meeting, 157
Barbadoes, 286
Book and its Story, A, 25, 38, 55, 92, 118, 135, 173, 211, 263, 288, 316, 350
Book World for Women and Nurses, The, 11
Broncho-Pneumonia in Children, 350
Butter, English, at a Kashmir Hospital, 301
Care and Education of the Feebleminded, The, 76
Cases of Interest, 157, 180
Central Bureau for Employment of Women, 161
Ceremony at the Curragh, 58
Change of Diet, A, 119
Charing Cross Hospital Bazaar, 174
Colonial Nursing Association, 238
Consumptives' Mode of Living at Davos, 344
Convalescent Fund, Our, 21, 42, 92, 281
Correction, A, 116, 270
Crusade of Cleanliness, A, 24
Dispensing Up-to-Date, 185
District Visiting Nursing in Obstetric Practice, 113, 127, 142
Doctor's Word Portrait of a Nurse, A, 283
"Don't," 103
Dorset County Nursing Homes, 12
Dying, Modes of, and Signs of Death, 86, 99
East-end Mothers' Home, The, 11
Echoes from the Outside World, 9, 19, 36, 53, 65, 78, 90, 104, 117, 132, 148, 159, 172, 186, 199, 212, 225, 240, 249, 262, 274, 287, 300, 314, 324, 335, 346
Egg Albumen in Sickness, The Use of, 248
Ethics of Nursing, The, 22, 37, 50
Everybody's Opinion, 12, 26, 42, 56, 68, 79, 93, 107, 121, 136, 149, 161, 175, 187, 201, 215, 226, 241, 252, 264, 276, 290, 302, 317, 327, 338, 352
Examination Questions, 67, 120, 175, 224
Exhibition of Nursing Requisites in Berlin, 129
Factory Girls' Country Holiday Fund, 160
Fête on Behalf of the Hammersmith Nurses, 213
First Aid Lectures in the Apennines, 270
Free Libraries and Anti-Vaccination, 21
French Hospitals, 323, 334
Greek Hospital, Alexandria, The, 311
Guy's Hospital, 37
Gynæcological Nursing, 155, 168, 180, 194, 206, 220, 232, 246, 258, 269, 281, 295, 308, 321
Hints on the Home Nursing of Sick Children, 3
History and Development of |Trained Nursing, The, 145, 158
Holiday Experiences, 282
Holidays for Overworked Nurses, 289
Home for Confirmed Invalids at Highbury, 63
Hospital Fête at Hampstead, 147
Hospital Johnnie, 272
Hospital Secrets, 285
Hospital for Sick Children, Nurses' House, 130
Hospital Sketches, 312, 348
Ideal Matron: What She Needs to be, The, 181
In a Hospital Garden, 283
In Memoriam—Louise Darche, 146
International Congress of Women Workers, The, 88, 122, 182, 197
Invalid Furniture, 39, 213
Legal, 10
Legal Intelligence, 133
Lichfield Victoria Nursing Home, 234
Life in a Plague Stricken District, 4
Lines to a Hospital Sister, 308
Lines to a "Mere Pro.," 336
London Hospital, The, 224
London School Nurses' Society, The, 170
Lunn's Tours, Dr., 82
Making Things Better, 62
Marriage of a Nurse, 261
Maternity Charity, Plaistow, The, 129
Matron's Corner, The, 33, 115, 147, 313, 349
Matron's Council, 196
Meath Home of Comfort, 241
Memorial Tablets at St. Thomas's Hospital, 208
Medical, Surgical, and Hygienic Exhibition Association, 106
Metropolitan Nursing Association, 64
Miss Burns' Wedding, 200
National Health Society, 147, 273
National Hospital for the Paralysed and Epileptic, The, 174
New Appointment, A, 175
Night Nursing Extraordinary, 286
Notes on News from the Nursing World, 1, 15, 29, 45, 59, 71, 83, 97, 111, 125, 139, 153, 165, 177, 191, 203, 217, 229, 243, 255, 267, 279, 293, 305, 319, 329, 341
Notes and Queries, 14, 28, 44, 58, 70, 82, 96, 110, 124, 138, 152, 162, 176, 190, 202, 216, 227, 242, 253, 265, 277, 291, 303, 318, 328, 339, 353
Novelties for Nurses, 11, 50, 91, 134, 190, 214
Nurse-Patient's Visit to Bad-Nauheim, A, 297
Nurse, The Born, 323
Nurse's Day of Rest, A, 143
Nurse's Experience Among the Mecca Pilgrims, A, 261
Nurse's Life in a Workhouse Hospital, A, 273
Nurse's Part in Sanitary Science, The, 10
Nurses, To, 34, 49, 64, 80, 96, 116, 135, 146, 155, 168, 184, 223, 263, 276, 284, 318
Nurses Amongst the Alps, 260
Nurses' Bookshelf, The, 37, 54, 79, 91, 289, 299, 313, 337
Nurses and County Council Lectureships, 119
Nurses at Marlborough House, The, 239
Nurses' Quarters at the Royal London Ophthalmic Hospital, The, 182
Nurses at Tracheotomy Drill, 326, 339
Nursing of Cases of Hernia, The,[331,[343
Nursing Fund Sunday, 241
Nursing Home at Pekin, A, 52
Nursing the Hop-pickers, 351
Nursing in the London Poor Law School Infirmaries, 315, 347
Nursing under Metropolitan Asylums Board, 34[illegible]
Nursing in New Zealand Hospitals, 101
Nursing on the Niger, 51
Nursing Organisation in New South Wales, 299
Nursing of Phthisis in Sanatoria under th[illegible] "Open-Air" System, The, 14, 128, 144, 156
Nursing Question in Italy, The, 75
Nursing Scarlet Fever Convalescents, 210
Nursing of Sick Prisoners in Scottish Prisons, 223
Nursing of Tracheotomy Cases, 209
Our Readers, To, 1
Paris Hospital, A, 298, 309
Pass It On, 334
Pension Fund Nurses, 14
Pictures from Italy, 169
Plague in India, 33[illegible]
Plague Nursing in India, A Year's, 183, 195, 207, 221, 233, 247, 259
Pocket Handkerchiefs in Illness, 86
Post Graduate Clinics for Nurses, 18, 34, 49
Practical Hygiene Teaching in Elementary Schools, 63
Princess Mary Village Homes, The, 20
Private Home for Ladies of Advanced Age, 185
Professional Pincushions, 40
Queen Charlotte's Nurses' Home, Opening of, 222
Queen Victoria's Jubilee Institute for Nurses, 196, 200
Raising the Wind at Penrith, 336
Royal British Nurses' Association, 77, 160, 198
Royal Infirmary, Preston, 251
Royal Isle of Wight Infirmary, Opening of a New Wing, 251
Royal Maternity Charity, The, 188
Royal National Pension Fund for Nurses, The, 102, 235
Russian Famine Fund, 174, 222
St. John's Hospital, Lewisham, 171
St. Mary's Hospital Bazaar, 143
St. Saviour's Homes, Hendon, 206
Sister's Post from Two Points of View, A, 250
Spring Novelties, 39
Suggestions for Private Nurses, 275
Suicide of a Nurse in a Railway Carriage, 214
Sussex County Hospital, The, 107
Swaffham Cottage Hospital, 283
Tommy: A Hospital Incident, 54
Training in Fever Hospitals, 311, 333
Travel Notes, 13, 27, 43 57, 69, 81, 95, 109, 123, 137, 151, 163, 189, 228, 254, 266, 278, 292, 304, 340, 354
Typhoid Fever and Typhoid Nursing, 32
Typical Patients, 41, 74, 88, 100, 115, 146, 171, 208, 275, 301
Unseen Cord, The, 271
"Ward No. 17," 150
Women's Total Abstinence Union, 68
Workhouse Infirmary Nursing, The Brighter Side of, 337
Workhouse Nursing, 105

Printed by W. H. and L. Collingridge, City Press, 148 and 149, Aldersgate Street, London.

**Notices of Births, Deaths, and Marriages** are inserted in this column at a charge of 2s. 6d. for an announcement not exceeding four lines.

### NOTICE TO CORRESPONDENTS.

All MS., Letters, Books for Review, and other matters intended for the Editor should be addressed The Editor, "The Hospital," The Hospital Building, 28 & 29, Southampton Street, Strand, W.C.

The Editor cannot undertake to return rejected MS., even when accompanied by stamped directed envelope.

**Notice to Advertisers and Subscribers.**

All Advertisements, Orders for Copies of The Hospital, and Business Communications should be addressed to **THE MANAGER** (**Not to the Editor**), The Hospital, The Hospital Building, 28 & 29, Southampton Street, Strand, London, W.C.

The Cost of Subscription, post free, is as follows:—Per week, 2½d.; per quarter, 2s. 8d.; per half-year, 5s. 3d.; per annum, 10s. 6d. Foreign:—Per annum, 15s. 2d., payable, in all cases, in advance.

**NOTICE.—Vol. XXV. of "The Hospital" (October, 1898, to March, 1899), in handsome cloth binding, will shortly be on sale at the Office of this Journal, price 7s. 6d. Cases for binding the Numbers contained in this Volume 1s. 6d. Index to Vol. XXV., 2½d., post free.**

**The Monthly Part for April is now ready. Price 6d., by post 8d.**

